# Changes in the Adrenal Cortex Induced by Liraglutide Treatment and Exercise in a Rat Model of Menopausal Transition

**DOI:** 10.3390/cells15141258

**Published:** 2026-07-13

**Authors:** Ivona Gizdović, Branka Šošić-Jurjević, Dragana Vlahović, Nataša Ristić, Irena Lavrnja, Branko Filipović, Ljiljana Marina, Svetlana Trifunović

**Affiliations:** 1 Institute for Biological Research “Siniša Stanković”—National Institute of Republic of Serbia, University of Belgrade, Bulevar Despota Stefana 142, 11108 Belgrade, Serbia; ivona.gizdovic@ibiss.bg.ac.rs (I.G.); brankasj@ibiss.bg.ac.rs (B.Š.-J.); dragana.vlahovic@ibiss.bg.ac.rs (D.V.); negicn@ibiss.bg.ac.rs (N.R.); irenam@ibiss.bg.ac.rs (I.L.); brankof@ibiss.bg.ac.rs (B.F.); 2 Center for Infertility and Endocrinology of Gender, University Clinical Center of Serbia, Faculty of Medicine, University of Belgrade, 11000 Belgrade, Serbia

**Keywords:** liraglutide, exercise, acyclic female rats, adrenal gland

## Abstract

The menopausal transition is a key period marked by changes in cardiometabolic health and adrenal function, representing an important window for targeted interventions to improve women’s health. Glucagon-like peptide-1 receptor agonists improve metabolic parameters, while physical exercise provides well-established benefits; however, their effects during the menopausal transition remain insufficiently explored. This study examined the effects of liraglutide (0.186 mg/kg; corresponding to the human equivalent dose of 1.8 mg/day used for type 2 diabetes treatment) and exercise on the adrenal gland in a rat model of menopausal transition. Three-month-old females served as young controls (CY), while 16-month-old acyclic females were assigned to control (C), liraglutide (L), exercise (E), or combined treatment (L+E). Compared with CY, the C group showed increased body mass and adrenal alterations, including reduced adrenal weight and volume, cortical atrophy, increased collagen content, decreased STAR, and increased pAMPKα optical density (*p* ≤ 0.05). *Sf1* and *Star* were upregulated in L, E, and L+E compared with C, most prominently in L (*p* ≤ 0.05), while *Cyp11b2* was increased in L and L+E (*p* ≤ 0.05). Hormone analysis showed reduced 17β-estradiol, corticosterone, and aldosterone in C compared with CY. Corticosterone was further reduced in L compared with C, while aldosterone increased in L and L+E compared with C (*p* ≤ 0.05). In conclusion, the menopausal transition induced adrenal morpho-functional remodeling. Liraglutide intervention had the greatest impact on steroidogenic output, both alone and in combination with exercise, while exercise alone showed no significant effect.

## 1. Introduction

The menopausal transition (MT) is characterized by metabolic, neuroendocrine, and vasomotor changes, with approximately 60–70% of women experiencing clinically relevant symptoms, largely associated with hormonal fluctuations [1]. Hormonal disturbances during this period trigger a cascade of health challenges, increasing cardiovascular and metabolic risks, accelerating bone loss, and causing a range of disruptive symptoms that significantly undermine women’s well-being and quality of life [2,3]. During the MT, fluctuations in reproductive hormones—particularly unstable estrogen levels— interfere with hypothalamic–pituitary–adrenal (HPA) axis activity, resulting in the dysregulation of hormone secretion within the axis [4]. Among the most well-documented changes during this period are alterations in cortisol regulation, reflected in a gradual age-related increase in morning cortisol levels and a progressive disruption of its circadian rhythm in humans [5,6]. In parallel, estrogen fluctuations modulate the renin–angiotensin–aldosterone system (RAAS), leading to altered aldosterone secretion from the adrenal cortex. This shift may contribute to fluid retention, changes in blood pressure, and increased cardiovascular risk as the protective effects of estrogen decline [7]. Hormone replacement therapy (HRT) is commonly used to alleviate metabolic and cardiovascular changes during the menopausal transition, but its use is limited in certain groups of women due to increased thromboembolic and long-term oncologic risks [8]. Glucagon-like peptide-1 receptor agonists (GLP-1RAs) have received widespread attention as effective pharmacological treatments for obesity and may offer additional benefits during the MT [9]. Initially developed as a pharmacological intervention for managing type 2 diabetes, GLP-1RAs have emerged as prominent anti-obesity therapies, achieving up to 15–20% weight loss. Beyond their effects on body weight and glycemic control, GLP-1RAs have demonstrated cardioprotective and bone-preserving effects [10], making them a promising intervention for age- and hormone-related health challenges, either alone or in combination with HRT. However, there are concerns regarding potential skeletal muscle loss associated with more powerful GLP-1RAs, such as semaglutide [11,12], an issue of particular importance in the context of aging and the menopausal transition. Liraglutide, although less potent in promoting weight loss, appears to have a more favorable profile for lean mass preservation, representing a safer therapeutic option for women during the MT [13,14]. Regular physical exercise is recognized as an effective strategy for health promotion and disease prevention during the MT [15], as it simultaneously addresses multiple menopausal symptoms. Recent evidence highlights the benefits of exercise in improving cardiometabolic status and physical fitness, including body weight, body mass index, cardiorespiratory fitness, and flexibility [16]. In addition, physical exercise supports skeletal muscle function and contributes to the maintenance of bone density during the MT [17]. Evidence from a single randomized controlled trial indicates that combining structured physical exercise with liraglutide reduces metabolic syndrome severity, abdominal obesity, and systemic inflammation, while improving physical performance and cardiorespiratory fitness beyond pharmacotherapy alone [18]. To date, while a few clinical studies examined physical exercise [19] or GLP-1 RAs [20] in postmenopausal women, the effects of these interventions during the menopausal transition itself remain underexplored. Given the adrenal glands’ central role in the regulation of metabolism and stress responses, their adaptation to GLP-1 RAs and physical exercise has received limited attention. Existing experimental studies of GLP-1 RAs [21,22] or physical exercise-induced adrenal adaptations [23,24] have been conducted predominantly in males, with females underrepresented or entirely excluded. This pronounced lack of data in females highlights an important knowledge gap and underscores the need for studies specifically addressing adrenal responses to GLP-1RAs and physical exercise in female subjects. This study aimed to evaluate the effects of GLP-1RA liraglutide and structured physical exercise on adrenal function and metabolic health in acyclic female rats as a model of the menopausal transition. In this study, 16-month-old acyclic female rats were selected because their progressive neuroendocrine shifts closely parallel the gradual trajectory of the human MT. Unlike surgical ovariectomy, which induces an abrupt, non-physiological loss of ovarian hormone production, this naturally aging model preserves the gradual, progressive endocrine decline characteristic of reproductive aging, providing a complementary framework for examining adrenal gland adaptations under liraglutide treatment [25]. This study consists of a detailed analysis of the adrenal gland, focusing on histomorphological changes in the zona glomerulosa (the principal site of aldosterone synthesis) and zona fasciculata (the principal site of corticosterone production), together with the expression of key steroidogenic genes, following liraglutide treatment and/or structured physical exercise.

## 2. Materials and Methods

### 2.1. Animals and Diets

This study included sixty female Wistar rats divided into two age groups: young adults (3 months old) and middle-aged animals (16 months old). Animals were maintained at the Unit for Experimental Animals, Institute for Biological Research “Siniša Stanković”—National Institute of the Republic of Serbia, under controlled environmental conditions (12 h light/12 h dark cycle, ambient temperature 21 ± 2 °C), with free access to food and water throughout the experiment. Females were fed a standard grain-based pellet diet (IG-Z-00117, Gebi d.o.o., Čantavir, Serbia).

### 2.2. Ethical Approval

All experimental procedures involving animals were conducted in accordance with the requirements of Directive 2010/63/EU for the protection of animals used for scientific purposes. Ethical approval for the study was granted by the Veterinary Directorate of the Ministry of Agriculture, Forestry and Water Management of the Republic of Serbia on 30 January 2024 (approval no. 000169446 2024 14841 002 001 000 001).

### 2.3. Experimental Design

**Figure 1:**
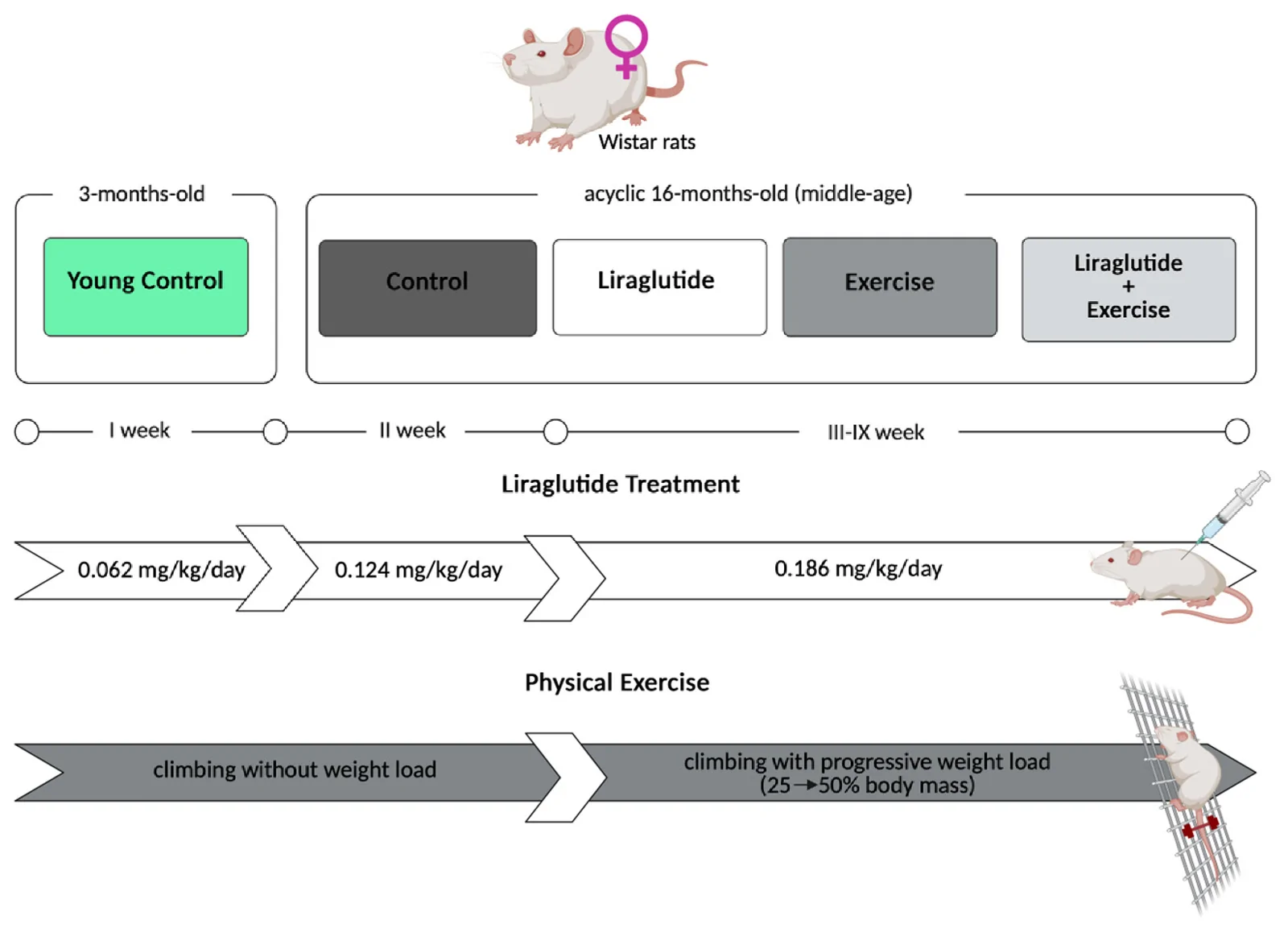
Female Wistar rats were divided into five experimental groups (*n* = 12 per group): young control (CY), middle-aged control (C), middle-aged liraglutide-treated (L), middle-aged exercise (E), and middle-aged liraglutide and exercise (L+E). Females in the liraglutide-treated groups (L and L+E) were administered daily subcutaneous injections of liraglutide (Saxenda^®^, Novo Nordisk A/S, Bagsværd, Denmark) at a final dose of 0.186 mg/kg body mass, dissolved in normal saline, with an injection volume of 0.3 mL. Treatment started at 0.062 mg/kg/day and gradually increased on a weekly basis until the final dose was achieved, which was maintained throughout the remaining 7 weeks. Controls (CY and C) and exercise (E) groups received an equivalent volume (0.3 mL) of normal saline. The exercise protocol consisted of a 2-week adaptation period involving ladder climbing without additional load, followed by a 7-week exercise period with progressively increased weight loads (25–50% of body mass). Created with BioRender. Gizdovic, I. (2026) https://BioRender.com/txeaj8d (accessed on 7 May 2026).

An overview of the experimental design is presented in Figure 1. The females were assigned to five groups (*n* = 12 per group): the first, young adult control (CY) group; the second, middle-aged control (C) group; the third, middle-aged liraglutide-treated (L) group; the fourth, middle-aged exercise (E) group; and the fifth, middle-aged liraglutide and exercise (L+E) group. Daily subcutaneous injections of liraglutide (Saxenda^®^, Novo Nordisk A/S, Bagsværd, Denmark) diluted in normal saline to a total volume of 0.3 mL were administered to the L and L+E groups. Liraglutide treatment started at a dose of 0.062 mg/kg/day (corresponding to the human equivalent dose of 0.6 mg/day). The dose was subsequently increased each week by 0.06 mg/kg until reaching the final dose of 0.186 mg/kg/day, corresponding to a human dose of 1.8 mg/day, which represents a submaximal therapeutic dose used in obesity treatment [26]. Liraglutide dosing was established through allometric inter-species scaling [27], while the treatment protocol, including a gradual weekly dose escalation during the adaptation phase, was developed in accordance with Saxenda^®^ prescribing guidelines (https://www.saxenda.com/about-saxenda/dosing-schedule.html, accessed on 13 December 2023). CY, C and E groups received 0.3 mL of normal saline. Treatments were administered once daily in the morning for nine weeks. Females receiving liraglutide were monitored throughout the treatment period, during which only mild gastrointestinal symptoms (mainly occasional diarrhea) were observed, with no other adverse effects. Females in the E and L+E groups underwent a physical exercise protocol based on vertical ladder climbing. The exercise apparatus consisted of a ladder (110 cm height, 2 cm grid spacing) positioned at an 80° incline, as previously described [28]. Females underwent a two-week acclimatization period of unweighted climbs. The subsequent seven-week training protocol comprised three sessions per week, during which weights were affixed to the tail base with adhesive tape, with the load beginning at 25% of body mass and gradually increasing up to 50%. Every training session consisted of repeated climbs from the base to the top of the ladder, with a 60 s recovery intervals between climbs, continuing until the animal could no longer complete the climb despite gentle tail stimulation. Each session was limited to a maximum duration of 10 min.

Performance during training was assessed through two parameters: the total quantity of successful climbs per session, reflecting training volume, and the load volume (LV), reflecting training intensity, calculated as: LV (g) = total quantity of successful climbs × weight load (g). Both parameters were averaged across the three sessions within a specific week, and the variation between the initial training week (25% body mass) and the final training week (50% body mass) was used for comparative analysis.

### 2.4. Estrous Cycle Monitoring

Vaginal cytology was performed daily throughout the monitoring period, with smears collected each morning to assess ovarian status. Based on the predominant cell types, estrous cycle stages were classified as proestrus, estrus, metestrus, and diestrus, as previously described [29]. Based on vaginal cytology analysis, young adult females exhibited regular estrous cyclicity throughout the monitoring period, whereas middle-aged females were acyclic. To ensure a reproductive phenotype consistent with the menopausal transition, only middle-aged rats exhibiting persistent estrus for at least 14 consecutive days prior to the intervention were included, with the status further confirmed by histological evaluation of the ovaries at the end of the experiment [30,31].

### 2.5. Glucose Tolerance Monitoring

To assess glucose tolerance, an intraperitoneal glucose tolerance test (ipGTT) was conducted five days prior to euthanasia, following an overnight fasting period. A glucose solution was administered intraperitoneally at a dose of 2 g/kg body mass and blood glucose was measured (GlucoSure AutoCode, Prizma, Kragujevac, Serbia) at 0, 15, 30, 60, 90, and 120 min post injection. The corresponding glucose area under the curve (AUC) was calculated using GraphPad Prism 8 software (v.8, San Diego, CA, USA).

### 2.6. Body Mass and Food Intake Measurements

Throughout the experimental period, body mass was measured on a weekly basis. The overall change in body mass was determined by calculating the difference between the values obtained at the end and the beginning of the experiment. In order to accurately assess individual food consumption, females were housed individually for a period of three days (including a one-day adaptation period) at the start of the experiment and again for three days (including a one-day adaptation period) a several days prior sacrifice.

### 2.7. Tissue Processing

Females were fasted overnight and humanely euthanized 24 h after the final treatment. Blood samples were collected, and serum and plasma were stored at −80 °C. Adrenal glands were excised, cleared of surrounding adipose tissue, and weighed. The left adrenal glands (*n* = 6 per group) were fixed in Bouin’s solution, processed through a graded ethanol dehydration series, cleared in xylene, and embedded in Histowax (Histolab Product Ab, Göteborg, Sweden). To perform histological, stereological, and immunohistochemical analyses, left adrenal gland tissue blocks were serially sectioned at 5 μm thickness using a rotary microtome (RM 2125RT, Leica Microsystems, Wetzlar, Germany). The right adrenal gland was snap-frozen in liquid nitrogen and stored at −80 °C for subsequent molecular analyses.

### 2.8. Histology and Stereology of Sirius Red and Novelli-Stained Adrenal Gland Sections

Adrenal sections were stained using Sirius Red and Novelli histological techniques as previously described [32]. In brief, Sirius Red staining was used to detect collagen fibers within the adrenal tissue, where type I collagen fibers appeared as red to red-orange structures. Novelli staining (acid fuchsin–light green) was applied to visualize the vascular profile of the tissue. This method enabled clear identification of erythrocytes, which appeared intensely purple within blood vessels and capillary networks, contrasting with the light-green background of the adrenal parenchyma. In addition to histological analysis, quantitative evaluation of connective and vascular tissue components was performed using stereological methods. Three adrenal sections per animal, obtained from the beginning, middle, and end of the serially sectioned gland, were analyzed at a final magnification of 20× using the newCAST software package (v. 2. 12. 1. 0; VIS—Visiopharm Integrator System, Visiopharm; Denmark). The reference area was defined using a mask tool, and a test grid with uniformly distributed points was applied on the tissue sections. On Sirius Red-stained sections, points hitting collagen fibers were recorded, while on Novelli-stained sections, points hitting erythrocyte accumulations within blood vessels were counted. The volume density (*V*_v_) was calculated using the following formula:

*V*_v_ (%) = (*P*_p_/*P*_t_) · 100

where *P*_p_ represents the number of points striking the structure of interest (collagen fibers or erythrocyte accumulations), and *P*_t_ represents the total number of points striking the reference area.

### 2.9. Histology and Stereology of Hematoxylin–Eosin-Stained Adrenal Gland Sections

Stereological analysis was performed on hematoxylin–eosin (H&E)-stained sections following histological evaluation. Adrenal gland sections were sampled in a systematic uniform random manner, beginning from a random starting point. Every 40th section from each tissue block was selected for analysis, resulting in a mean distance of 200 µm between two consecutively analyzed sections. A test grid with 25 sampling points (arranged in a 5 × 5 matrix) was superimposed on each section. To assess the accuracy of the volume estimates, the coefficient of error (CE) was calculated using a standard shape factor (alpha = 4) appropriate for the organ’s geometry. The mean CE across all analyzed animals was 0.051 (ranging from 0.042 to 0.068), confirming high methodological reliability and sampling precision well within the acceptable stereological threshold. The newCAST stereological software package (version 2. 12. 1. 0; VIS—Visiopharm Integrator System, Visiopharm; Denmark) was used for analysis. The volumes of the adrenal gland, cortical zones and medulla were estimated using Cavalieri’s principle [33]. The volumes were calculated according to the following formula:

$$V=a(p)\cdot BA\cdot \sum_{i=1}^{n}Pi$$

where a(p) represents the area associated with a single sampling point, BA (the block advance) is the mean distance between two consecutively analyzed sections, ΣPi is the total number of points hitting the structure of interest, and n is the number of sections studied for each adrenal gland.

The individual cell volume in the zona glomerulosa and fasciculata was estimated using the planar rotator as an unbiased local estimator [33]. For each animal, between 150 and 200 cells with visible nuclei were analyzed per zone (zona glomerulosa and zona fasciculata). Measurements were performed at an objective magnification of 100×.

### 2.10. Histology ImageJ-Based Quantitative Analysis of STAR and phosphoAMPKα-Immunostained Adrenal Gland Sections

Following deparaffinization and rehydration, the adrenal gland sections underwent antigen retrieval in 0.1 M citrate buffer (pH 6.0) via microwave heating (750 W, 5 min), and then the sections were left to cool at room temperature for 45 min. Following this step, endogenous peroxidase activity was blocked with 0.3% hydrogen peroxide in methanol for 15 min. Nonspecific binding was reduced by incubation with normal swine serum (1:10; X0901, Dakopatts, Glostrup, Denmark) for 1 h at room temperature. Subsequently, sections were incubated with rabbit polyclonal anti-STAR antibody (1:100; orb7014, Biorbyt, Cambridge, UK) or rabbit polyclonal anti-phospho-AMPKα (Thr172) antibody (1:100; #2531, Cell Signaling Technology, Danvers, MA, USA) for two nights at room temperature. After washing in PBS, incubation with HRP-conjugated anti-rabbit IgG (1:100; PO399, Dakopatts, Glostrup, Denmark) was performed for 1 h. Immunoreactivity was visualized using 3,3′-diaminobenzidine chromogen system (Dako North America, Inc. Carpinteria, CA, USA), followed by counterstaining with hematoxylin. Sections were then dehydrated, cleared, and mounted in DPX medium (Sigma-Aldrich, Barcelona, Spain). Stained section images were captured with a Leica DM4 B microscope (Leica Microsystems CMS GmbH, Wetzlar, Germany) using the Leica Application Suite X (LAS X) software, version 3.8.1.26810. STAR and pAMPKα immunostaining intensity within the adrenal gland were measured via the FIJI ImageJ software (version 21. 0. 7; National Institutes of Health, Bethesda, MD, USA). For each animal, eight non-overlapping micrographs (960 × 720 pixels, 40× magnification) covering the entire adrenal gland section were analyzed, with entire micrographs considered as regions of interest. All images were acquired under identical illumination and exposure settings and converted to 8-bit grayscale prior to analysis. IHC signal was quantified using the IHC Profiler plugin. This tool automatically classified pixel intensity and calculated optical density (OD), which reflected the amount of deposited chromogen and provided semi quantitative measure of staining intensity.

### 2.11. Adrenal Gland Gene Expression Analyses

Total RNA was isolated from adrenal tissue using TRIzol reagent (Invitrogen, Carlsbad, CA, USA) according to the manufacturer’s instructions. The concentration of RNA was determined by measuring OD at 260nm using Nanophotometer^®^ N60 (IMPLEN, Munich, Germany) at 260 nm, while RNA purity was assessed by OD_260_/OD_280_ and OD_260_/OD_230_ ratios. An amount of 1 µg of total RNA was reverse-transcribed into cDNA using a High-Capacity cDNA Reverse Transcription Kit (Applied Biosystems, Vilnius, Lithuania) in total reaction volume of 20 µL. Quantitative real-time PCR (qRT–PCR) was conducted on a QuantStudioTM 3 Real-Time PCR System (Applied Biosystems, Waltham, MA, USA) utilizing the SYBR^®^ Green PCR Master Mix (Bio-Rad Laboratories, Hercules, CA, USA). All samples were evaluated in duplicate. Target gene expression levels were determined using the comparative 2^−∆Ct^ method, with *Hprt* serving as the reference gene. *Hprt* was selected as the most stable reference gene based on a systematic evaluation of candidate housekeeping genes, demonstrating the highest expression stability and minimal intergroup variability across experimental conditions.

The sequences for all primers are provided in Supplementary Table S1.

### 2.12. Biochemical Analyses

Biochemical analyses were performed using serum and plasma obtained from blood collected at the time of sacrifice. For plasma preparation, blood was collected in EDTA-containing tubes and kept at 4 °C until processing. Serum and plasma samples were centrifuged at 3000× *g* for 15 min, and the supernatants were aliquoted and stored at −80 °C for subsequent biochemical and hormonal analyses.

Serum concentrations of sodium (Na+), potassium (K+), chloride (Cl−), and inorganic phosphorus (Pi) were determined via a DIESTRO 103AP V4 S+ semi-automatic electrolyte analyzer (JS Medicina Electrónica SRL, Buenos Aires, Argentina). Plasma concentrations of lactate, lactate dehydrogenase (LDH), creatine kinase (CK), total cholesterol, HDL-C, LDL-C, non-HDL-C, and triglycerides were evaluated with a BS-240 Vet Chemistry Analyzer (Shenzhen Mindray Animal Medical Technology Co., Ltd., Shenzhen, China) following the manufacturer’s protocol. Serum estradiol levels were determined using an electrochemiluminescence immunoassay (ECLIA) on the Roche diagnostic platform (Roche Diagnostics, Mannheim, Germany; Ref. No. 06656021190), according to the manufacturer’s instructions. The detection limits of the assay spanned from 5 to 3000 pg/mL. Serum aldosterone levels were measured using a commercially available enzyme-linked immunosorbent assay (ELISA) kit (Demeditec Diagnostics GmbH, Kiel, Germany, Cat. No. DE5298), following the manufacturer’s protocol. The assay detection range spanned from 0 to 1000 pg/mL. Plasma corticosterone concentrations were determined with a Rat/Chicken CORT ELISA Kit (Elabscience Biotechnology Co., Ltd., Wuhan, China, Cat. No. E-EL-0160) following the manufacturer’s protocol. The detection limits of the assay spanned from 0.39 to 25 ng/mL. The assay detection range spanned from 0.39 to 25 ng/mL. Plasma insulin concentrations were quantified using a commercial RIA kit (INEP, Belgrade, Serbia; sensitivity: 0.6 mIU/L; intra- and inter-assay CVs: 2.5% and 7.7%).

### 2.13. Statistical Analysis

Statistical analyses were performed using GraphPad Prism software (v.8, San Diego, CA, USA). The normality of data distribution was assessed using the Shapiro–Wilk test. When data followed a normal distribution, differences between groups were analyzed using one-way ANOVA, with Tukey’s post hoc test applied for multiple comparisons. For data that failed to meet the normality assumption, the non-parametric Kruskal–Wallis test, followed by Dunn’s post hoc test was performed. Body mass and food intake differences between the start and end of the experiment were assessed by two-way repeated-measures ANOVA with Sidak’s multiple comparisons test applied subsequently. The level of statistical significance was defined as * *p* ≤ 0.05, ** *p* ≤ 0.01, *** *p* ≤ 0.001, and **** *p* ≤ 0.0001. All values are presented as group mean ± standard error of the mean (SEM).

## 3. Results

### 3.1. Body Mass, Food Intake, Adrenal Gland Weight, and Glycemic Control

**Figure 2:**
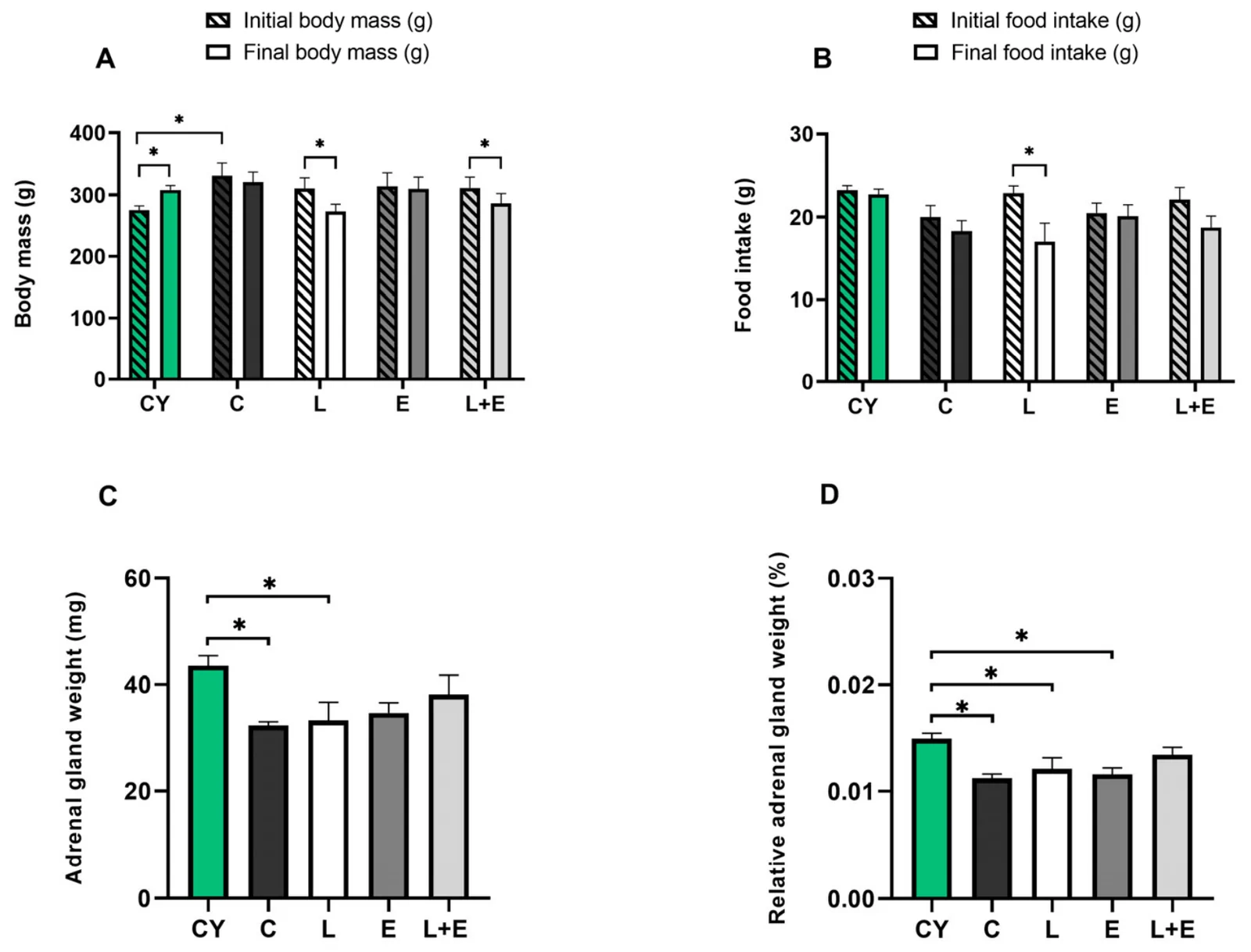
Body mass (**A**), food intake (**B**), absolute (**C**), and relative (**D**) adrenal gland weights in female rats across experimental groups: young control (CY), middle-aged control (C), middle-aged liraglutide-treated (L), middle-aged exercise (E), and middle-aged liraglutide and exercise (L+E). Data are presented as mean ± SEM (*n* = 12 per group). Baseline body mass differences between CY and C groups were analyzed using an unpaired two-tailed *t* test. Changes in body mass and food intake over the experimental period were analyzed using two-way repeated measures ANOVA followed by Sidak’s post hoc test for pairwise comparisons. Changes in absolute and relative adrenal gland weight were analyzed using one-way ANOVA followed by Tukey’s post hoc test. * *p* ≤ 0.05.

The C group showed a 20% higher (*p* ≤ 0.05) initial body mass compared with the CY group. A two-way repeated measures ANOVA revealed a significant Treatment × Time interaction (*p* ≤ 0.0001, η^2^p = 0.46) demonstrating that the interventions (L, L+E) differentially affected body mass changes over the two-month experimental period. Body mass significantly increased (*p* ≤ 0.05) in the CY group, while it decreased in the L and L+E groups (Figure 2A). Food intake was monitored throughout the study. A two-way repeated measures ANOVA revealed a significant main effect of Time (*p* = 0.0047, η^2^p = 0.196), whereas the Treatment × Time interaction did not reach statistical significance (*p* = 0.1375, η^2^p = 0.168). To further investigate the effect of time, separate analyses within each group were performed. This revealed a significant reduction in food intake over time exclusively within the L group (*p* ≤ 0.05), while intake in the other groups remained stable (Figure 2B). Absolute adrenal gland weight was significantly decreased (*p* ≤ 0.05) in the C (26%) and L (24%) groups compared with the CY group (Figure 2C). Relative adrenal gland weight was significantly reduced (*p* ≤ 0.05) in the C (17%), L (20%) and E (17%) groups compared with the CY group (Figure 2D).

**Figure 3:**
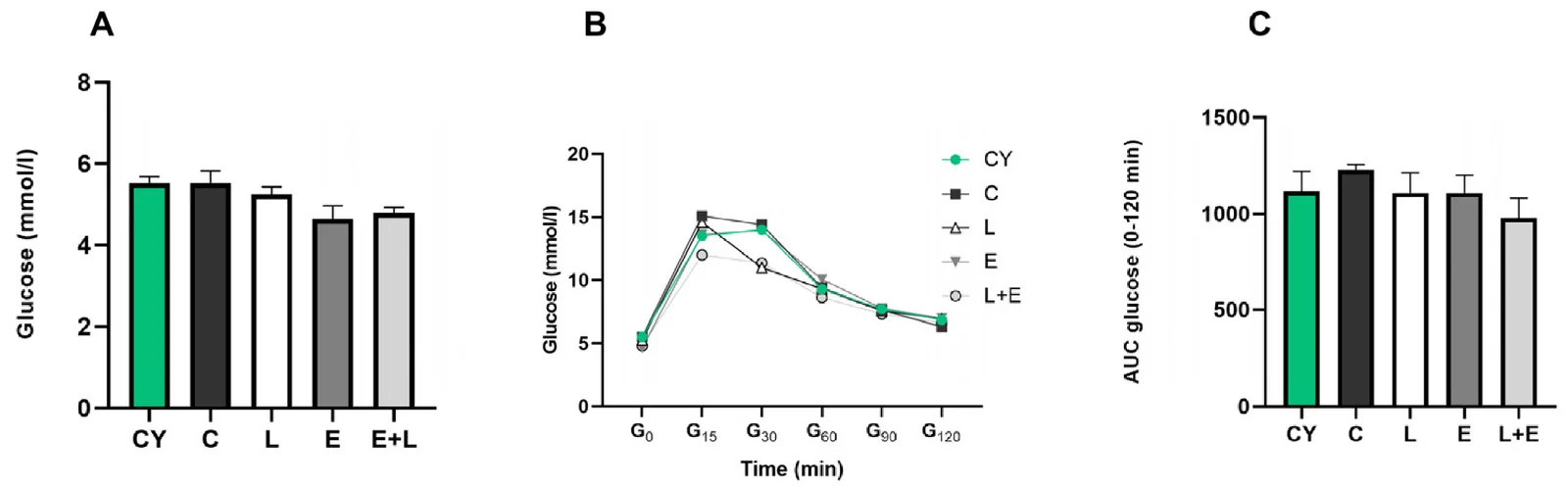
Fasting blood glucose levels (**A**), intraperitoneal glucose tolerance test curve (**B**), and area under curve (**C**) in females across experimental groups: young control (CY), middle-aged control (C), middle-aged liraglutide-treated (L), middle-aged exercise (E), and middle-aged liraglutide and exercise (L+E). Data are presented as mean ± SEM (*n* = 12 per group) and were analyzed by one-way ANOVA followed by Tukey’s post hoc test.

Fasting blood glucose levels (Figure 3A) did not differ significantly among the experimental groups. Glucose response curves obtained during the ipGTT (Figure 3B) showed similar dynamics across all groups, with comparable curve profiles and AUC values (Figure 3C). Fasting plasma insulin levels did not differ significantly among the experimental groups (CY: 21.7 ± 1.6; C: 20.3 ± 2.9; L: 20.6 ± 1.7; E: 20.9 ± 1.9; L+E: 19.3 ± 1.9 mlU/L, one-way ANOVA, *p* = 0.9494).

### 3.2. Training Performance, Biochemical and Electrolyte Markers

In the E group (*n* = 12 per group), the number of successful climbs increased from 3.5 ± 0.2 per session at 25% body mass load to 4.7 ± 0.5 per session at 50% body mass load, corresponding to an initial LV rising from 281.8 ± 27.4 g to a final LV of 715.7 ± 86.3 g, representing a significant increase (*p* ≤ 0.001). Similarly, in the L+E group (*n* = 12 per group), the number of successful climbs increased from 3.3 ± 0.4 to 4.0 ± 0.5 per session, corresponding to an initial LV rising from 266.3 ± 53.8 g to a final LV of 601.6 ± 110.5 g, which also represented a significant increase (*p* ≤ 0.01). No significant differences were observed between the final LVs of the E and L+E groups. Exercise-related parameters, including lactate, LDH, and CK, remained unchanged among the experimental groups (Table 1, Exercise-related parameters).

Regarding the lipid profile, plasma non-HDL-C levels were significantly increased (*p* ≤ 0.05) in the C and L+E groups compared to the CY group, while triglycerides were significantly elevated (*p* ≤ 0.05) only in the C group compared to the CY (Table 1, Lipid profile). Serum Na^+^ and Cl^−^ levels remained unchanged across all experimental groups, whereas K^+^ levels showed a decreasing trend in the C group compared with the CY group; this reduction was more pronounced (*p* ≤ 0.05) in the L+E group relative to the CY group. P_i_ levels were significantly elevated (*p* ≤ 0.05) in the L compared with the E group (Table 1, electrolytes).

### 3.3. Histological and Stereological Analysis of Sirius Red- and Novelli-Stained Adrenal Gland Sections

Gross histological analysis revealed clearly preserved adrenal gland zonation in all groups, with no visible pathological alterations. The adrenal cortex exhibited the typical architecture consisting of the zona glomerulosa (ZG, cells arranged in compact, rounded clusters), zona fasciculata (ZF, elongated cells organized in radially oriented cords), and zona reticularis (ZR, a network of smaller cells surrounding blood vessels) beneath a connective tissue capsule containing collagen fibers.

**Figure 4:**
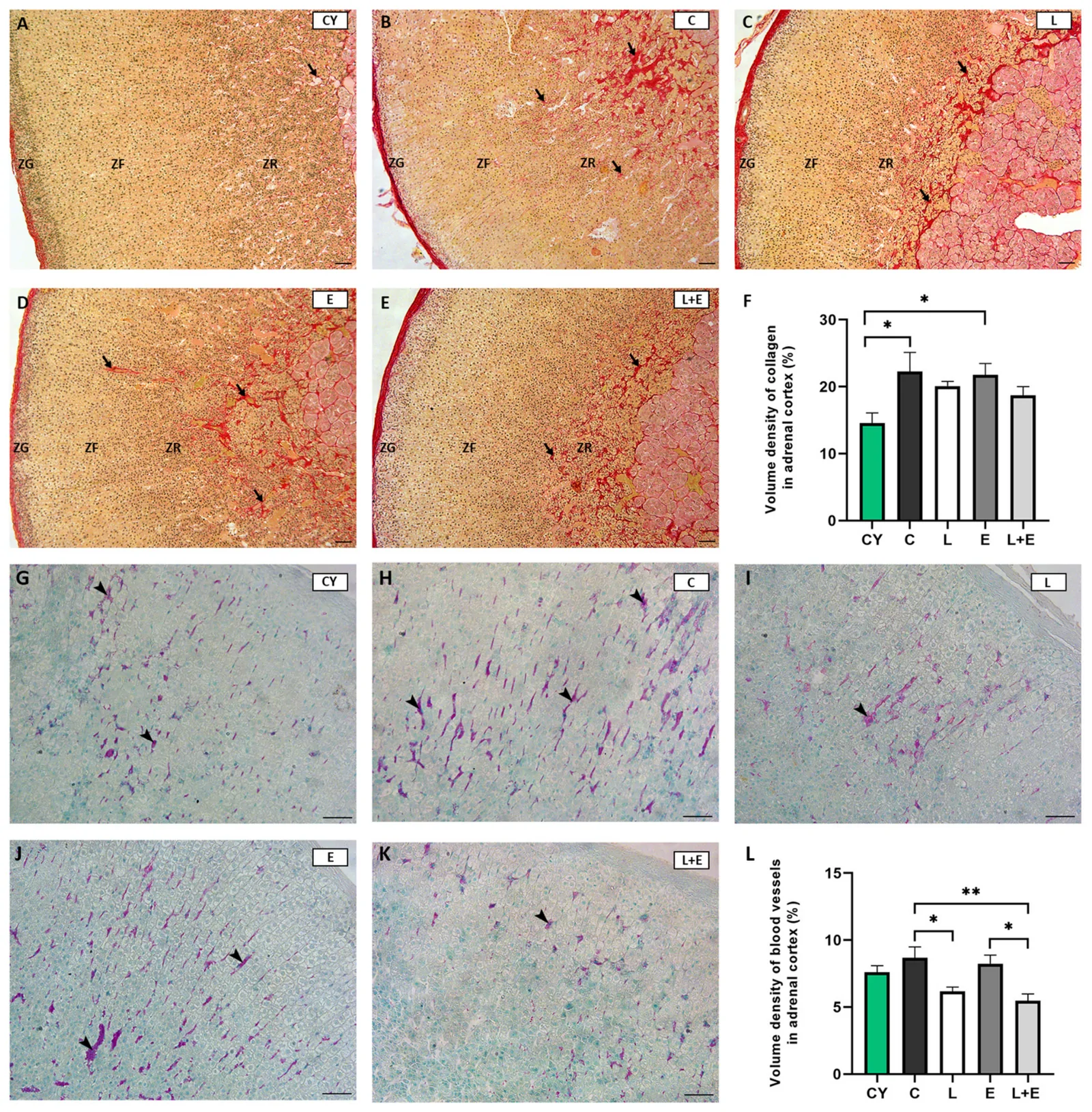
Sirius Red- (**A**–**E**) and Novelli-stained (**G**–**K**) sections of the adrenal gland in female rats across experimental groups: young control (CY), middle-aged control (C), middle-aged liraglutide-treated (L), middle-aged exercise (E), and middle-aged liraglutide and exercise (L+E). Representative micrographs show the zona glomerulosa (ZG), zona fasciculata (ZF), zona reticularis (ZR), collagen (arrows), and blood vessels (arrowheads); scale bar = 50 µm (**A**–**E**, **G**–**K**). Volume density of collagen (**F**) and blood vessels (**L**) in the adrenal cortex across the experimental groups. Data are presented as mean ± SEM (*n* = 6 per group) and analyzed by one-way ANOVA followed by Tukey’s post hoc test. * *p* ≤ 0.05, ** *p* ≤ 0.01.

Histological analysis revealed increased collagen content (fibrosis) in the adrenal glands of middle-aged groups compared with the CY group, as demonstrated by Sirius Red staining, which specifically highlights collagen fibers in the adrenal capsule and stromal (interstitial) regions (Figure 4A–E). Based on stereological measurements, the volume density of collagen fibers in the adrenal gland increased in all middle-aged groups, with a significant increase (*p* ≤ 0.05) in the C and E group compared with the CY group (Figure 4F).

Greater vascularization was observed in the L and L+E groups, as demonstrated by Novelli staining, which enabled clear visualization of the adrenal vasculature, with purple-stained blood vessels and capillary networks sharply contrasting with the green-stained adrenal tissue (Figure 4G–K). Stereological analysis confirmed the histological findings, demonstrating a significant reduction (*p* ≤ 0.05) in the volume density of blood vessels in the L and L+E groups compared with the C group (Figure 4L).

### 3.4. Histological and Stereological Analysis of Hematoxylin–Eosine Stained Adrenal Gland Sections

**Figure 5:**
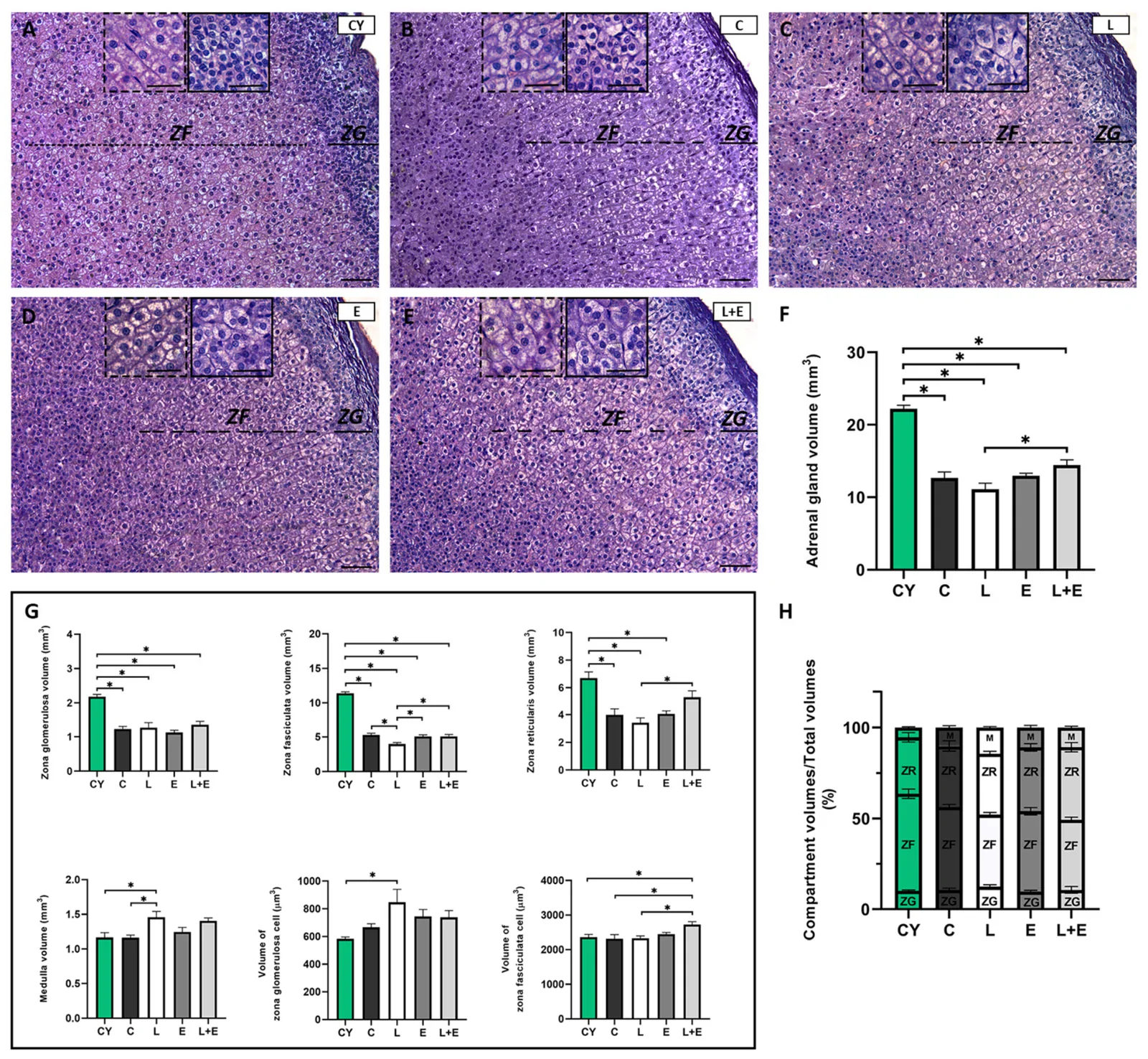
Hematoxylin–eosin-stained sections (**A**–**E**) of the adrenal gland in female rats across experimental groups: young control (CY), middle-aged control (C), middle-aged liraglutide-treated (L), middle-aged exercise (E), and middle-aged liraglutide and exercise (L+E). Representative micrographs show the zona glomerulosa (ZG) and zona fasciculata (ZF). Boxed areas indicate the regions magnified in the inserted micrographs corresponding to the ZF (left) and ZG (right); scale bar = 50 µm (**A**–**C**), scale bar = 25 µm inserted micrographs. Volume of the adrenal gland (**F**), volumes of cortical zones and medulla, as well as volumes of individual cells in the zona glomerulosa and zona fasciculata (**G**), and volume density of adrenal gland compartments (**H**) across the experimental groups. Data are presented as mean ± SEM (*n* = 6 per group) and analyzed by one-way ANOVA followed by Tukey’s post hoc test. * *p* ≤ 0.05.

Histological analysis of sections of the adrenal glands revealed an age-related decrease in the thickness of the cortical zones, with the most pronounced reduction in the zona fasciculata (Figure 5A–E). Similar to the reduction in adrenal weight with aging, stereological analysis showed a significant age-associated decrease in adrenal gland volume. A significant reduction (*p* ≤ 0.05) was observed in the C, L, E, and L+E groups compared with the CY group, by 43%, 50%, 42%, and 35%, respectively (Figure 5F). Stereological measurements showed that the largest individual cell volumes were found in the ZG of the L group and in the ZF of the L+E group, respectively (Figure 5G). Despite age-related reductions in the absolute volume of the adrenal gland and its individual zones, the relative proportions of these zones within the gland remain unchanged (Figure 5H).

### 3.5. Histological and ImageJ Analysis of STAR and Phospho-AMPKα-Immunostained Adrenal Gland Sections

**Figure 6:**
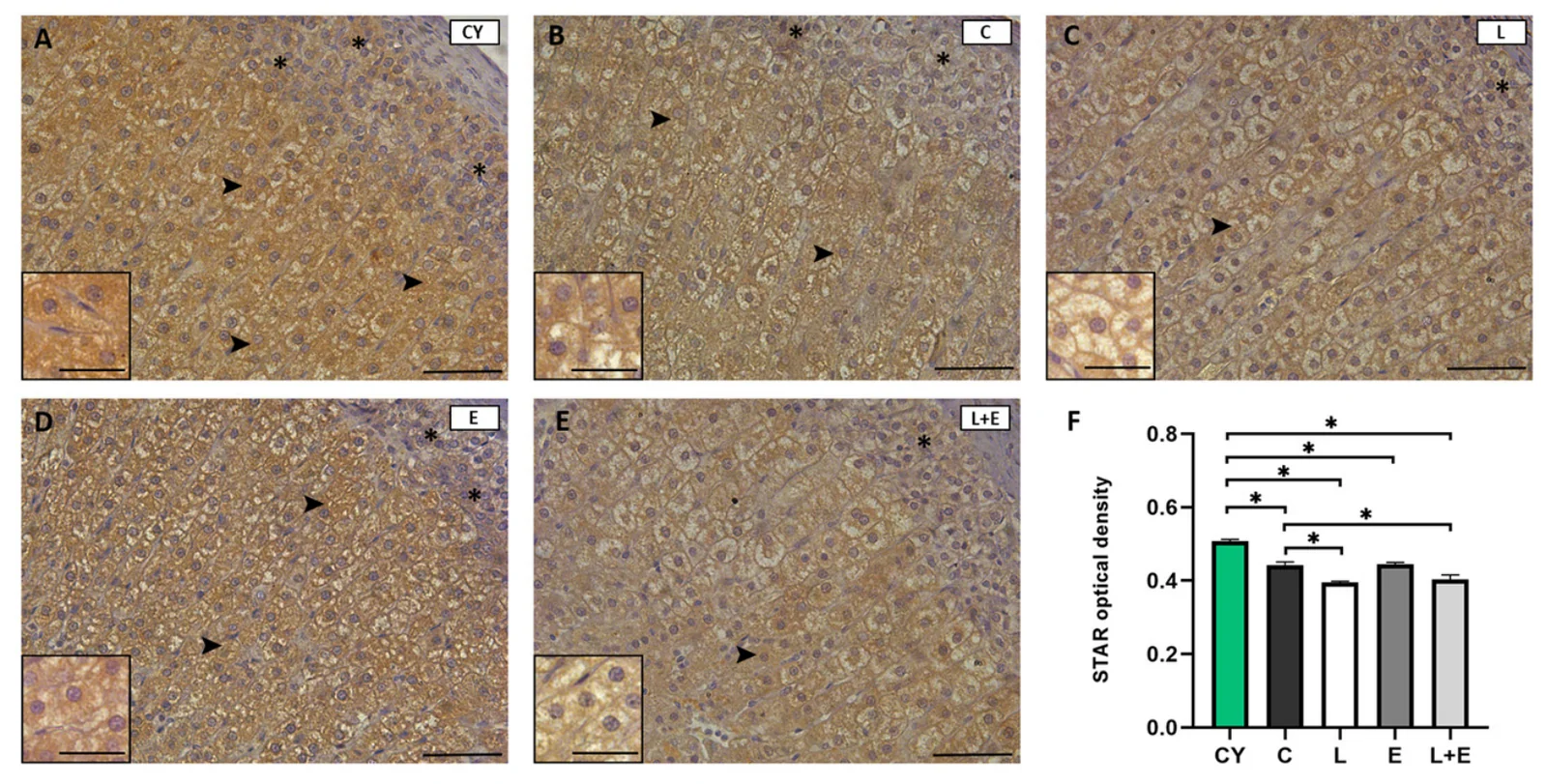
STAR immunoreactivity in the adrenal gland in female rats across experimental groups: young control (CY), middle-aged control (C), middle-aged liraglutide-treated (L), middle-aged exercise (E), and middle-aged liraglutide and exercise (L+E). Representative micrographs (**A**–**E**) show STAR immunoreactivity in the zona glomerulosa (asterisks) and zona fasciculata (arrowheads); scale bar = 50 µm, scale bar = 25 µm inserted micrographs. Quantitative analysis of STAR optical density (**F**) in the adrenal cortex across the experimental groups. Data are presented as mean ± SEM (*n* = 6 per group) and were analyzed by one-way ANOVA followed by Tukey’s post hoc test. * *p* ≤ 0.05.

Immunohistochemical evaluation of STAR protein expression in the adrenal cortex of all experimental groups showed a broad distribution across all three zones. However, all middle-aged groups exhibited reduced immunopositivity compared to the young control group. A higher proportion of cells with absent or low STAR immunopositivity was found in the L and L+E groups compared with the C group (Figure 6A–E). Quantitative analysis of immunohistochemical staining revealed significant differences in STAR optical density among the experimental groups. The CY group exhibited the highest STAR optical density compared with all middle-aged groups (*p* ≤ 0.05) L and L+E groups showed a further decreased optical density compared with the C group (*p* ≤ 0.05).

**Figure 7:**
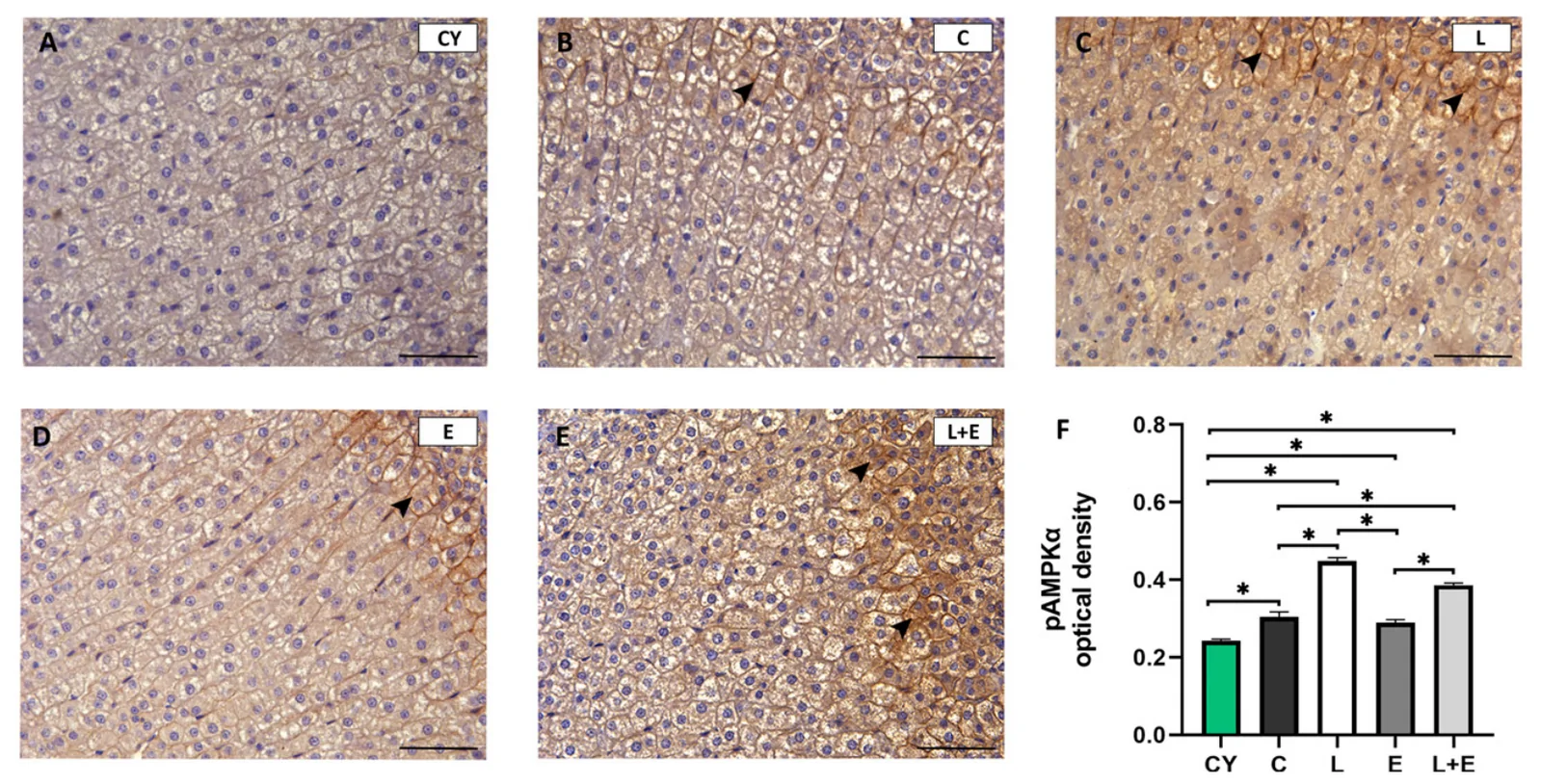
Phospho-AMPKα immunoreactivity in the adrenal gland in female rats across experimental groups: young control (CY), middle-aged control (C), middle-aged liraglutide-treated (L), middle-aged exercise (E), and middle-aged liraglutide and exercise (L+E). Representative micrographs (**A**–**E**) show phospho-AMPKα immunoreactivity in the adrenal cortex (arrowheads indicate areas of positive immunostaining); scale bar = 50 µm. Quantitative analysis of phospho-AMPKα optical density (**F**) in the adrenal cortex across the experimental groups. Data are presented as mean ± SEM (*n* = 6 per group) and were analyzed by one-way ANOVA followed by Tukey’s post hoc test. * *p* ≤ 0.05.

Immunohistochemical evaluation of phospho-AMPKα protein expression in the adrenal cortex revealed stronger immunopositivity in all middle-aged groups compared with the young control group (Figure 7A–E). Furthermore, quantitative analysis of phospho-AMPKα immunohistochemical staining showed significantly higher optical density in the L and L+E groups than in the CY, C, and E groups (*p* ≤ 0.05).

### 3.6. Expression of Genes of Interest in the Adrenal Gland

**Figure 8:**
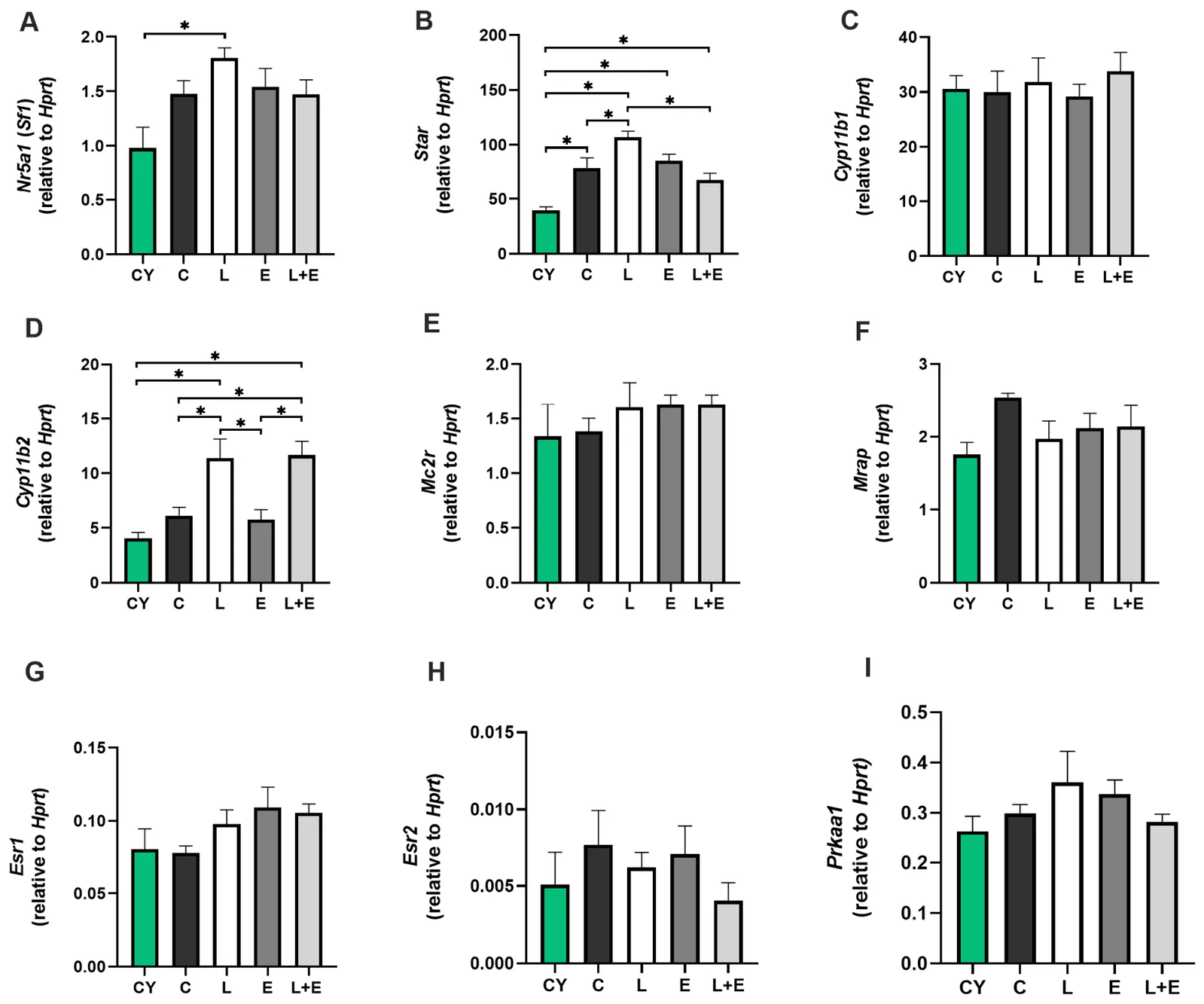
mRNA expression of *Nr5a1* (**A**), *Star* (**B**), *Cyp11b1* (**C**), *Cyp11b2* (**D**), *Mc2r* (**E**), *Mrap* (**F**), *Esr1* (**G**), *Esr2* (**H**), and *Prkaa1* (**I**) in female rats across experimental groups: young control (CY), middle-aged control (C), middle-aged liraglutide-treated (L), middle-aged exercise (E), and middle-aged liraglutide and exercise (L+E). Data are presented as mean ± SEM, *n* = 8 per group. For normally distributed data, statistical significance was determined by one-way ANOVA followed by Tukey’s post hoc test; for non-normally distributed data, the Kruskal–Wallis followed by Dunn’s post hoc test was applied. * *p* ≤ 0.05.

The expression of *Nr5a1*, which encodes steroidogenic factor 1 (SF1)—a key regulator of adrenal development and steroidogenic enzyme expression—was higher in all middle-aged groups compared with the CY group. This increase was statistically significant (*p* ≤ 0.05) in the L group compared with the CY group (Figure 8A). Similarly, *Star* expression, which mediates mitochondrial cholesterol transport and represents the rate-limiting step in steroidogenesis, was significantly higher (*p* ≤ 0.05) in all middle-aged groups with the CY group. Moreover, *Star* expression was significantly increased (*p* ≤ 0.05) in the L group compared with the C group (Figure 8B). The expression of *Cyp11b1*, which encodes the enzyme responsible for converting 11-deoxycorticosterone to corticosterone, remained unchanged across all groups (Figure 8C). In contrast, *Cyp11b2*, which encodes the enzyme that catalyzes the conversion of 11-deoxycorticosterone to aldosterone, showed higher expression in all middle-aged groups than in the CY group. This increase was statistically significant (*p* ≤ 0.05) in the L and L+E groups compared with both the CY and C groups (Figure 8D). *Mc2r* expression, which encodes the ACTH receptor (melanocortin 2 receptor, MC2R), was modestly elevated compared with the CY group, but this was not statistically significant (Figure 8E). The expression of *Mrap*, which encodes an accessory protein essential for MC2R trafficking to the cell membrane and efficient ACTH signaling, showed a slight age-related increase; however, this change did not reach statistical significance (Figure 8F). Although a slight increase in the expression of *Esr1* and *Esr2*, encoding estrogen receptor α and estrogen receptor β, respectively, was observed, these changes were not statistically significant. (Figure 8G,H). The expression of *Prkaa1*, which encodes the catalytic α1 subunit of AMPK, was slightly increased in the middle-aged groups compared with the CY group; however, the difference was not statistically significant (Figure 8I).

### 3.7. Blood Hormone Levels

Serum levels of 17β-estradiol were lower in all middle-aged groups compared to the CY group; however, due to pronounced individual variability, this decrease reached statistical significance (*p* ≤ 0.05) only between the CY and C groups. Similarly, plasma corticosterone showed a significant age-related decline (*p* ≤ 0.05), with a further reduction observed in the L and L+E groups compared with the C group; however, statistical significance was reached only between the L and C groups with corticosterone levels 30% lower in the L group. Serum aldosterone levels were significantly reduced (*p* ≤ 0.05) in the C group compared with the CY group (by 44%). Conversely, both the L and L+E treatments significantly increased (*p* ≤ 0.05) aldosterone levels by 57% and 48%, respectively, compared with the C group (Table 2).

## 4. Discussion

The menopausal transition, a period of significant endocrine imbalance that affects whole-body function, is associated with adverse health outcomes, and represents a critical window for therapeutic interventions. This study investigated a rat model of the menopausal transition (acyclic middle-aged female rats), focusing on adrenal morpho-functional characteristics considering previously inconclusive data, and evaluated the effects of experimental interventions, including chronic liraglutide treatment and structured physical exercise.

An age-related increase in body mass was observed in middle-aged compared with young control rats despite unchanged food intake. Circulating levels of 17β-estradiol, corticosterone, and aldosterone were lower in middle-aged females. At the adrenal level, decreases in adrenal weight and volume, together with reductions in cortical zonal volumes and an increase in collagen content, were noted. STAR protein expression within the ZG and ZF showed weaker and more diffuse immunopositivity in middle-aged females, despite a slight upregulation of genes involved in steroidogenesis. The increase in body mass and unchanged food intake may reflect an age-related decline in energy expenditure and basal metabolic rate [34], consistent with broader patterns of age-related metabolic dysfunction and shifts in body composition observed in rodent models [35,36,37]. During reproductive aging in rat models, progressive ovarian senescence, and follicular depletion lead to acyclicity and a shift toward persistent estrus, resulting in fluctuating and ultimately reduced estrogen levels [31]. Reductions in adrenal hormone levels are largely attributed to estrogen-dependent mechanisms, given the significant regulatory influence of estrogen on both the RAAS [38] and the HPA axis [39]. However, findings across studies regarding hormone levels remain inconsistent, due to the pronounced and dynamic endocrine fluctuations characteristic of menopausal transition [5,40]. The reduction in adrenal zonal volumes (hypoplasia of adrenal cortical zones) together with decreased STAR protein expression, is associated with lower adrenal hormone levels [41]. In contrast, increased expression of steroidogenic genes represents a potential compensatory transcriptional response to impaired adrenal function. However, transcriptional upregulation does not necessarily translate into increased steroid output due to post-transcriptional and mitochondrial regulatory constraints. Reduced mitochondrial cholesterol transport may limit glucocorticoid synthesis, indicating a dissociation between gene expression and functional steroidogenesis, which is supported by the increased phospho-AMPKα protein expression observed in this study [42].

Chronic liraglutide treatment (submaximal therapeutic dose for obesity) in middle-aged females led to reduced body mass and food intake. Circulating corticosterone levels decreased, while aldosterone levels increased, with no changes in 17β-estradiol. At the adrenal gland level, liraglutide had no effect on gland weight or overall volume, and zonal organization was preserved. However, a tendency towards reduced collagen content and vascularization was observed, along with a slight increase in cell size within the ZG. STAR protein expression was markedly decreased, particularly in the ZF. The expressions of *Sf1*, *Star*, and *Cyp11b2* (which catalyzes the conversion of 11-deoxycorticosterone to aldosterone) were upregulated, while expression of *Cyp11b1* (responsible for the conversion of 11-deoxycorticosterone to corticosterone) remained unchanged.

As liraglutide is widely used as a therapeutic agent for weight reduction [43], the observed decrease in body weight, although expected, validates our animal model by confirming that the selected dosage and treatment duration were effective. This is relevant as the study focuses on the adrenal gland, rather than the well-characterized GLP-1RAs’ target organs. GLP-1RAs act centrally by modulating brain regions involved in appetite and energy balance via neurotransmitters and peptides, and peripherally by improving glycemic control through enhanced insulin secretion, reduced glucagon release, and delayed gastric emptying, ultimately leading to weight loss [44].

Liraglutide treatment significantly increased plasma aldosterone levels, potentially via a mechanism linking renal GLP-1R activation to increased *Cyp11b2* expression and aldosterone synthesis within the RAAS. GLP-1 receptors are localized within the renal vasculature, specifically in the vascular smooth muscle cells of arteriolar and arterial regions [45] and their activation has been shown to induce *Ren1* gene expression [46], thereby stimulating the renin-angiotensin cascade and increasing angiotensin II availability. Angiotensin II subsequently acts on the adrenal zona glomerulosa to upregulate *Cyp11b2* transcription [47]. An indirect, RAAS-mediated pathway is supported by the absence of GLP-1R expression across all three zones of the adrenal cortex in rodents and humans [48,49] with its restriction to the adrenal medulla [21,50], though interspecies variations require caution in cross-species interpolation [49]. The absence of GLP-1R in human zona glomerulosa (H295R) cells [51] additionally supports an indirect, RAAS-mediated mechanism. The regulatory effect of liraglutide on aldosterone secretion is highly time-dependent, characterized by a suppression of circulating levels during acute administration but an elevation following chronic exposure [52,53]. Given the increased *Star* gene expression yet decreased StAR protein expression, alongside elevated phospho-AMPKα protein levels, and the rise in *Cyp11b2* gene expression and aldosterone blood levels observed in this study, we suppose aldosterone production may involve both StAR-dependent and StAR-independent mechanisms acting at the level of aldosterone biosynthesis [54].

Corticosterone levels were decreased in our study, which contrasts with the majority of research conducted primarily on the male sex [21,55,56], where GLP-1RAs stimulate the HPA axis via receptors in the paraventricular nucleus [57]. Clinical data also show that long-term GLP-1RAs exposure in healthy individuals does not necessarily activate the HPA axis [58]. This discrepancy highlights that males and females show significantly different responses. Specifically, a recent clinical study using receptor radiolabeling demonstrated that males exhibit significantly higher uptake of radiolabeled exendin in the pituitary gland, reflecting a higher density of GLP-1 receptors compared to females [59]. This difference correlates with a sex-specific ACTH response, suggesting that HPA axis sensitivity is modulated by biological sex [60] and hormonal phases [61]. In contrast to central stimulation, studies on isolated adrenocortical cells demonstrate that GLP-1 can directly inhibit ACTH-induced corticosterone secretion [62]. The decreased StAR and increased phospho-AMPKα protein expression, despite the observed increase in *Star* mRNA expression reported here, support the post-transcriptional regulation of corticosterone synthesis. In the context of liraglutide use during the menopausal transition, these findings may be considered clinically favorable, suggesting a metabolically stabilizing rather than a stress-related response.

In the present study, physical exercise in middle-aged females did not significantly alter adrenal morpho-functional parameters and resulted in modest, non-significant changes in circulating corticosterone, aldosterone, and 17β-estradiol levels. This lack of a standalone effect may be attributed to specific factors regarding exercise modality, duration, and intensity within an aging female framework. Regarding exercise modality, we applied a resistance training protocol (ladder climbing); while aerobic training primarily induces sustained, volume-driven stress on the HPA axis [63], resistance training stimulates the axis through acute, high-intensity tension followed by rapid recovery intervals [64]. This recovery dynamic is further reflected in our exercise intensity markers, as plasma lactate levels remain stable during structured ladder climbs due to interval recovery [65] being consistent with our non-significant end-point lactate differences, confirming a stable metabolic baseline rather than an acute response. Regarding duration, the 7-week training period represents a chronic stimulus, while acute resistance exercise transiently spikes corticosterone [65] long-term training leads to neuroendocrine habituation and a blunted basal adrenal response [66,67]. Crucially, the literature in aging female populations indicates that the aging HPA axis exhibits decreased sensitivity and altered feedback control [68]. Therefore, the combination of a resistance modality, interval-based intensity, and a 7-week duration likely allowed the already less-sensitive aging HPA axis to completely habituate to the workload, maintaining overall HPA axis stability while potentially enhancing adaptive responsiveness to subsequent stress [24,69]. Similarly, acute physical exercise stimulates aldosterone secretion via renin–angiotensin–aldosterone system (RAAS) activation [70], whereas chronic physical exercise attenuates this response, resulting in reduced basal RAAS activity [71]. In line with these findings, our study also showed a decrease in aldosterone levels following chronic physical exercise, although this change did not reach statistical significance. Finally, while physical exercise is traditionally associated with a reduction in circulating estrogen levels—primarily through the loss of adipose tissue and decreased aromatase activity—recent evidence and our current findings suggest that chronic physical exercise can also lead to a slight, non-significant increase in 17β-estradiol levels, likely mediated by stimulated adrenal androgen production and shifts in sex hormone-binding globulin levels [72,73].

Similar to the L group, females in the L+E group showed a significant reduction in body mass, indicating that the observed weight loss was mainly driven by liraglutide and was not substantially modified by the addition of physical exercise, suggesting a dominant pharmacological effect at the systemic metabolic level. At the hormonal level, the L+E group showed a trend toward reduced circulating corticosterone and a significant increase in aldosterone levels, reflecting the effects observed in the L group. This indicated that these changes are primarily driven by liraglutide and are maintained under the combined intervention.

## 5. Conclusions

In conclusion**,** our study demonstrates that reproductive aging during the menopausal transition induces substantial adrenal morpho-functional remodeling. Liraglutide treatment emerged as the primary driver of this adrenal morpho-functional remodeling, completely overriding the effects of exercise, with these tissue-level changes ultimately resulting in a distinct hormonal dissociation characterized by reduced corticosterone and significantly elevated aldosterone production. Conversely, resistance training alone exerted no measurable impact on these evaluated adrenal parameters in this model. Translationally, these findings bridge a critical gap in male-centered literature by characterizing the female adrenal cortex during the menopausal transition, while underscoring the clinical necessity of monitoring adrenal function during liraglutide therapy. As a pioneering study on the combined effects of liraglutide and exercise during the menopausal transition, certain limitations are present; namely, a deeper exploration of zone-specific intracellular signaling pathways and a comprehensive characterization of treatment-induced shifts in systemic appetite regulators—such as ghrelin—are required to fully elucidate the underlying pathways, thereby establishing crucial avenues for our upcoming mechanistic trials.

## Figures and Tables

**Table 1:** Plasma exercise-related parameters, lipid profile, and serum electrolyte levels in young control (CY), middle-aged control (C), middle-aged liraglutide-treated (L), middle-aged exercise (E), and middle-aged liraglutide and exercise (L+E) groups of females.

	CY	C	L	E	L+E
Exercise-related parameters					
Lactate (mmol/L)	5.6 ± 0.80	8.5 ± 1.19	6.98 ± 1.04	6.02 ± 0.67	6.92 ± 0.41
LDH (U/L)	2308 ± 276.5	2444 ± 356.3	1903 ±251.1	1932 ± 87.60	2085 ± 331.9
CK (U/L)	2663 ± 272.2	2293 ± 264.8	3017 ± 97.98	2930 ± 218.6	2549 ± 256.0
Lipid profile					
Total cholesterol (mmol/L)	1.64 ± 0.15	1.99 ± 0.14	1.81 ± 0.09	1.83 ± 0.19	2.01 ± 0.15
HDL-C (mmol/L)	0.47 ± 0.05	0.42 ± 0.04	0.49 ± 0.06	0.41 ± 0.04	0.51 ± 0.06
LDL-C (mmol/L)	0.33 ± 0.05	0.81 ± 0.21	0.55 ± 0.07	0.51 ± 0.05	0.55 ± 0.18
Non-HDL-C (mmol/L)	1.03 ± 0.08	1.48 ± 0.12 ^CY^	1.42 ± 0.05	1.31 ± 0.14	1.55 ± 0.12 ^CY^
Triglycerides (mmol/L)	0.77 ± 0.05	1.09 ± 0.09 ^CY^	0.82 ± 0.06	1.02 ± 0.06	0.85 ± 0.09
Electrolytes					
Na^+^ (mmol/L)	144.18 ± 0.73	142.8 ± 0.75	143.0 ± 0.99	142.9 ± 1.11	141.7 ± 0.68
K^+^ (mmol/L)	5.28 ± 0.99	4.57 ± 0.22	5.13 ± 0.26	5.25 ± 0.12	4.27 ± 0.12 ^CY,L,E^
Cl^−^ (mmol/L)	101.5 ± 0.68	101.4 ± 0.49	100.8 ± 0.53	101.9 ± 0.96	101.3 ± 0.37
P_i_ (mmol/L)	3.31 ± 0.20	2.70 ± 0.13	3.69 ± 0.48	2.33 ± 0.11 ^L^	2.78 ± 0.22

Data are presented as mean ± SEM (*n* = 12 per group). Statistical significance was determined by one-way ANOVA followed by Tukey’s post hoc test. Significant differences were considered at *p* ≤ 0.05 and indicated with respect to the corresponding groups (CY, L, E).

**Table 2:** Serum aldosterone and estradiol, and plasma corticosterone in young control (CY), middle-aged control (C), middle-aged liraglutide (L), middle-aged exercise (E), and middle-aged liraglutide and exercise (L+E) groups of females.

	CY	C	L	E	L+E
17β-estradiol (pg/mL)	57.92 ± 5.75	30.98 ± 4.55 ^CY^	34.56 ± 5.32	39.03 ± 9.32	40.60 ± 6.80
Corticosterone (ng/mL)	60.49 ± 6.44	39.52 ± 3.68 ^CY^	27.58 ± 1.63 ^CY,C^	36.44 ± 1.24 ^CY^	30.56 ± 1.83 ^CY^
Aldosterone (ng/dL)	16.76 ± 1.62	9.44 ±0.72 ^CY^	14.79 ± 1.24 ^C^	8.79 ± 0.82 ^CY,L^	14.01 ± 0.99 ^C,E^

Data are presented as mean ± SEM (*n* = 12 per group). Statistical significance was determined by one-way ANOVA followed by Tukey’s post hoc test for normal distributed data and by Kruskal–Wallis with Dunn’s post hoc test for non-normal distributed data. Significant differences were considered at *p* ≤ 0.05 and indicated with respect to the corresponding groups (CY, C, L, E).

## Data Availability

The datasets used and/or analyzed during the current study are available from the corresponding author on reasonable request.

## Supplementary Materials

The following supporting information can be downloaded at: https://www.mdpi.com/article/10.3390/cells15141258/s1, Table S1: Primer sequences for RT—qPCR.
