# Correction: Gizdović et al. Changes in the Adrenal Cortex Induced by Liraglutide Treatment and Exercise in a Rat Model of Menopausal Transition. *Cells* 2026, *15*, 1258

**DOI:** 10.3390/cells15151386

**Published:** 2026-07-31

**Authors:** Ivona Gizdović, Branka Šošić-Jurjević, Dragana Vlahović, Nataša Ristić, Irena Lavrnja, Branko Filipović, Ljiljana Marina, Svetlana Trifunović

**Affiliations:** 1 Institute for Biological Research “Siniša Stanković”—National Institute of Republic of Serbia, University of Belgrade, Bulevar Despota Stefana 142, 11108 Belgrade, Serbia; ivona.gizdovic@ibiss.bg.ac.rs (I.G.); brankasj@ibiss.bg.ac.rs (B.Š.-J.); dragana.vlahovic@ibiss.bg.ac.rs (D.V.); negicn@ibiss.bg.ac.rs (N.R.); irenam@ibiss.bg.ac.rs (I.L.); brankof@ibiss.bg.ac.rs (B.F.); 2 Center for Infertility and Endocrinology of Gender, University Clinical Center of Serbia, Faculty of Medicine, University of Belgrade, 11000 Belgrade, Serbia; ljikka@yahoo.com

## Addition of an Author

In the original publication [1], Ljiljana Marina was not included as an author. Dr. Marina’s critical role in the early conceptual and design stages of the project was inadvertently overlooked during the compilation of the final author list. Upon a subsequent collective review and discussion among all contributors, it was unanimously agreed that her foundational intellectual input satisfies the journal’s strict authorship criteria. An additional affiliation was added accordingly.

The corrected Author Contributions statement appears here. The authors state that the scientific conclusions are unaffected. This correction was approved by the Academic Editor. The original publication has also been updated.

**Author Contributions:** Conceptualization: B.Š.-J., L.M. and S.T.; design of the study: B.Š.-J., L.M., S.T. and B.F.; animal experiments: I.G., D.V., B.Š.-J., N.R. and S.T.; methodology, formal analysis, data curation and interpretation: I.G., D.V., B.Š.-J., N.R., I.L., L.M. and S.T.; writing—original draft preparation: I.G. and S.T.; writing—review and editing: S.T. and I.G.; visualization: I.G. and S.T.; funding acquisition: B.F. All authors have read and agreed to the published version of the manuscript.
