# Supplementary material for: Changes in the Adrenal Cortex Induced by Liraglutide Treatment and Exercise in a Rat Model of Menopausal Transition
Source: Cells. 2026 Jul 13;15(14):1258. doi: 10.3390/cells15141258 (PMC13406854; doi:10.3390/cells15141258)
Supplement: Supplementary file 1 [file cells-15-01258-s001.zip › cells-4333718-supplementary.pdf]

Supplementary Table S1. Primer sequences for RT – qPCR

Gene	Primer Sequences (5′-3′)	Accession Number
Nr5a1	F: ATGGCGGACCAGACCTTTATC<br>R: GCTGTCTTCCTTGCCGTACTG	NM_001191099.1
Star	F: AGCAAGGAGAGGAAGCTATGC<br>R: GGCACCACCTTACTTAGCACT	NM_031558.3
Cyp11b1	F: AGAGTATCCTCCCGCATCG<br>R: GCCAGTCTGCCCCATTTAG	NM_012537
Cyp11b2	F: TGGAGATGTGTGTGCCAAAT<br>R: CTTCCACATGGGGTCTGTCT	NM_012538.2
Mc2r	F: CGCTACATCACCATCTTCCA<br>R: CCGCTCCCTGTACAGAACAT	NM_001100491.1
Mrap	F: ATTCTGCTCTACATGTCCTG<br>R: TACTTCCTGGCTCATCTG	NM_001135834.1
Esr1	F: GCGCAAGTGTTACGAAGTGG<br>R: AGTGCCCATTTCATTTCGGC	NM_012689.1
Esr2	F: GTTGTGCCAGCCCTGTTACT<br>R: ACATGACCAAACGCCGTAAT	NM_012754.1
Prkaa1	F: GGGCGGGTGAAGATCGG<br>R: AGTCAACTCGTGCTTGCCCA	NM_019142.3
Hprt	F: CAGTCCCAGCGTCGTGATTA<br>R: AGCAAGTCTTTCAGTCCTGTC	NM_012583.2
